# Whole-genome sequence and annotation of *Penstemon davidsonii*

**DOI:** 10.1093/g3journal/jkad296

**Published:** 2023-12-29

**Authors:** Kate L Ostevik, Magdy Alabady, Mengrui Zhang, Mark D Rausher

**Affiliations:** Department of Evolution, Ecology, and Organismal Biology, University of California Riverside, Riverside, CA 92521, USA; Department of Biology, Duke University, Durham, NC 27708, USA; Department of Plant Biology, University of Georgia, Athens, GA 30602, USA; Department of Statistics, University of Georgia, Athens, GA 30602, USA; Department of Biology, Duke University, Durham, NC 27708, USA

**Keywords:** *Penstemon davidsonii*, Davidson's beardtongue, PacBio Hifi, Hi-C, genome assembly, genome annotation

## Abstract

*Penstemon* is the most speciose flowering plant genus endemic to North America. *Penstemon* species’ diverse morphology and adaptation to various environments have made them a valuable model system for studying evolution. Here, we report the first full reference genome assembly and annotation for *Penstemon davidsonii*. Using PacBio long-read sequencing and Hi-C scaffolding technology, we constructed a de novo reference genome of 437,568,744 bases, with a contig N50 of 40 Mb and L50 of 5. The annotation includes 18,199 gene models, and both the genome and transcriptome assembly contain over 95% complete eudicot BUSCOs. This genome assembly will serve as a valuable reference for studying the evolutionary history and genetic diversity of the *Penstemon* genus.

## Introduction

*Penstemon* (Plantaginaceae) is a genus made up of many flowing plant species (∼280) commonly known as the beardtongues. It is the largest plant genus endemic to North America. *Penstemon* species have diverse vegetative and floral morphology and are adapted to a wide range of environments (Nold 1999; Wolfe *et al*. 2006; Wilson *et al*. 2007; Thomson and Wilson 2008). This diversity has led to *Penstemon* emerging as a model system for the study of evolution, especially with regard to pollination syndromes (e.g. Wilson *et al*. 2004; Wessinger *et al*. 2014; Katzer *et al*. 2019), speciation (e.g. Straw 1955; Clark 1971; Wolfe *et al*. 1998; Stone *et al*. 2023), and repeated evolution (e.g. Wilson *et al*. 2007; Wessinger and Rausher 2014).

In addition to several chloroplast genomes (Ricks *et al*. 2017, Stettler *et al*. 2021), 2 nuclear genomes have recently become available for Penstemon, *P. barbatus* (Wessinger *et al*. 2023) and *P. kunthii* (Schlenk 2023). However, additional reference genomes for species in the genus would significantly enhance our understanding of *Penstemon's* biology and open up new avenues of research, such as studies of chromosomal evolution and comparative genomics.

*Penstemon davidsonii* E. Greene is a perennial species that occurs in the alpine zone of the Sierra Nevada and Cascade Mountain ranges. It is a diploid with 8 chromosomes and a predicted genome size of 483 Mb (Broderick *et al*. 2011). *P. davidsonii* occurs within subgenus Dasanthera (Datwyler and Wolfe 2004; Wolfe *et al*. 2021), which is the outgroup to the rest of *Penstemon*. Although partial genome sequences have previously been generated for this species (Dockter *et al*. 2013), a full reference genome for subgenus Dasanthera is needed. This will allow researchers to infer the ancestral genome for this clade and, therefore, better understand evolutionary changes that occurred within *Penstemon*.

Here, we report the first full genome assembly for *P. davidsonii*. We use a combination of PacBio and Hi-C sequencing to assemble a de novo reference genome for this species.

## Materials and methods

### Study organism and collection

We collected cuttings from multiple *P. davidsonii* plants along Piute Pass in the Sierra Nevada Mountain range in June 2016. These cuttings were allowed to root in water, transferred to a 1:1 mix of potting soil and pumice, and kept under controlled conditions in the Duke University greenhouse. Because *P. davidsonii* hybridizes with *P. newberryi* along Piute Pass (Clausen *et al*. 1940, Chabot and Billings 1972, Datwyler and Wolfe 2004, Kimball 2008), we used population genetic data from Garcia *et al*. (2023) and phenotypic characteristics to identify an individual with little *P. newberryi* ancestry. Of the plants collected, DNT005 appeared to be exclusively *P. davidsonii*. This individual was collected at 11,598 ft (latitude: 37.2422, longitude: −118.68265) and used for subsequent sequencing.

### DNA isolation and PacBio sequencing

We finely ground flash-frozen leaf tissue from DNT005 using a mortar and pestle and extracted DNA using a Cetyltrimethyl Ammonium Bromide (CTAB) protocol (Clarke 2009) with the following modifications. After grinding tissue, we performed 3 washes with a 0.35 M sorbitol buffer to remove secondary metabolites before cell lysis (Inglis *et al*. 2018). We added 2% polyvinylpyrrolidone, 2% dithiothreitol, and 2% beta-mercaptoethanol to the CTAB buffer because each of these reagents has been shown to improve DNA extraction in difficult plant tissues (e.g. Porebski *et al*. 1997, Horne *et al*. 2004, Aboul-Maaty and Oraby 2019). Finally, we incubated samples with RNAse A for 30 min at 37°C before precipitating DNA to remove RNA from the samples.

We purified the DNA using AMPure beads (Beckmann Coulter, USA) and assessed quality using a Large Fragment Analysis Kit (Agilent, USA). The library was prepared using the SMRTbell Express Template Prep Kit 2.0 (Pacific Biosciences, USA) and Sequel II Binding Kit 2.0 with a peak insert size of ∼18 kbp (mean = 12,986 bp). The sequencing was performed on the Sequel II system with a movie time of 30 h. This yielded 3,829,617 reads with a mean length of 30,922. The SMRTbell library and sequencing were done at the Georgia Genomics and Bioinformatics Core.

### Hi-C library preparation and sequencing

We used intact leaf tissue from DNT005C and the Proximo TM Hi-C kit (Phase Genomics, USA) to generate libraries for Hi-C sequencing. In brief, nuclear DNA was crosslinked before endonuclease treatment with DpnII, and the fragmented dsDNA was biotinylated to create junctions as per Phase genomics protocols (van Berkum *et al*. 2010). These biotinylated fragments were purified and subjected to paired-end Illumina 2 × 150 sequencing yielding 141.6 million read pairs.

### Genome assembly

The genome was assembled using HiCanu v2.1 (Nurk *et al*. 2020) consensus sequences and duplicate purged using “purge_dups” (Guan *et al*. 2020) to reduce the assembly to haplotype as much as possible. Then, we used the 3D-DNA pipeline (Dudchenko *et al*. 2017) with the default values for scaffolding. Specifically, we calculated the enzyme restriction sites for DpnII with respect to the draft assembly from HiCanu using the script generate_site_positions.py provided by the Juicer (Durand *et al*. 2016) toolkit. This restriction site map was used by the Juicer toolkit when aligning the Hi-C sequences against the draft assembly using bwa (Li 2013) to generate a duplicate-free list of paired alignments that were used by the 3D-DNA pipeline during scaffolding.

NextPolish (Hu *et al*. 2020) is a high-accuracy genome polishing tool that can incorporate both short reads and Pacbio HiFi reads. We prepared our reads for use by NextPolish as follows. The Hi-C reads were trimmed using cutadapt (Martin 2011) to remove low-quality bases and Ns at the start of reads. The HiFi reads were generated from the Pacbio subreads.bam using the Pacbio CCS14 tool. Then, we instructed the NextPolish pipeline to use bwa for the short read mapping and minimap2 in the mode designed for Pacbio CCS genomic reads for the HiFi read mapping. The specific modifications we made to the NextPolish configuration file are as follows: (1) the lgs_option section was removed as the raw Pacbio subreads were not being used, (2) a hifi_option section was added and set to “- min_read_len 1k -max_depth 100,” and (3) the hifi_minimap2_options section was set to “-x asm20.” The completeness of the assembly was assessed using BUSCO v4.0.6. (Simão *et al*. 2015) based on evolutionarily informed expectations of gene content from the eudicot ODB10 database. Finally, we used the Foreign Contamination Screening tool from the National Center for Biotechnology Information (Sayers *et al*. 2023) to identify contaminating sequences in the final assembly. This revealed no sequences from unintended organisms and only 4 small regions (20–30 bp) of adaptor contamination, which we subsequently replaced with Ns.

### RNA-seq and transcriptome assembly

We extracted RNA from frozen roots, leaves, and whole seedlings of the DNT005C plant using a Spectrum Plant Total RNA Kit (Sigma-Aldrich, USA). Libraries were made using the KAPA Stranded mRNA-Seq Kit (Roche, Switzerland) with NEBNext Multiplex Oligos (96 Unique Dual Index Primer Pairs) for Illumina Barcodes (New England Biolabs, USA) and sequenced on an Illumina NovaSeq 6000 S2 150 bp PE flow cell at the Duke Center for Genomic and Computational Biology Sequencing and Genomic Technologies Core.

The RNA-seq short reads were trimmed and quality filtered using trim_galore (Krueger 2015). All cleaned paired and unpaired reads were de novo assembled using Trinity v2.6.6 (Grabherr *et al*. 2011) with default parameters (Haas *et al*. 2013) to generate the transcriptome. We used BUSCO in the transcriptome mode to estimate transcriptome completeness.

### Genome annotation and masking

We identified and masked sequence repeats through the following steps. First, we identified tandem repeats within the genome using Tandem Repeat Finder v4.09 with specific parameter settings, including matching weight = 2, mismatching penalty = 5, indel penalty = 7, match probability = 0.8, indel probability = 0.1, minimum alignment score = 50, and maximum period size = 2,000 (Benson 1999). Second, we identified interspersed repeats and LTR retrotransposons using RepeatModeler v2.0.2 (Flynn *et al*. 2020). For the LTR transposons, we used the LTRStruct flag in RepeatModeler. Next, the identified repeat families were clustered using CD-HIT-EST v4.8.1 with 90% sequence identity and a seed size of 8 bp (Fu *et al*. 2012). Finally, the generated repeat library was applied to mask interspersed repeat elements in the assembly and develop repeat annotation files using RepeatMaskerv4.1.0 (Smit *et al*. 2015).

To prepare for gene annotation using the MAKER pipeline (Campbell *et al*. 2014), we took the following steps. The cleaned paired RNA-seq reads were mapped to the genome assembly using Tophat v2.1.2 followed by gene annotation using Cufflinks v2.2.1. We predicted tRNAs in the final assembly using tRNAscan-SE v2.0.7 with the default parameters (Chan and Lowe 2019). To identify miRNA precursor genes, we first retrieved 38,589 miRNAs precursors from the miRBase database (Griffiths-Jones *et al*. 2008) and clustered them separately to remove redundancies using CD-HIT-EST v4.8.1 (Fu *et al*. 2012), resulting in a total of 25,760 sequences. Then, these nonredundant sequences were used to identify the miRNA precursors in the *Penstemon* genome using homology-based search by BLASTN alignment tool with the default thresholds (Altschul *et al*. 1990).

We used the soft-masked genome generated using RepeatMasker v4.1.0 with the Repbase repeat library and all the previous annotations (i.e. TE, RNA-seq, miRNA, and tRNA) for gene prediction in the MAKER pipeline. Evidence from ab initio and homology-based methods were integrated to perform the final gene predictions. Specifically, the EST evidence from the RNA-seq assembly and ab initio gene predictions of the genome were used to construct the gene set using the MAKER pipeline. AUGUSTUS v3.2.3 (Stanke and Morgenstern 2005) was used for the ab initio gene prediction, and the BLAST alignment tool was used for homology-based gene prediction using the EST evidence in the MAKER pipeline. Exonerate v2.4.0 (Slater and Birney 2005) was used to polish and curate the BLAST alignment results. And, we manually removed partial CDSs for 35 genes.

## Results and discussion

Our PacBio sequencing produced 117.5 Gb (3.8 million reads with a mean length of 30,922), which represents 268× average coverage of our 438 Mb final genome (Table 1). This genome size is 91% of the 483 Mb estimate based on flow cytometry (Broderick *et al*. 2011).

**Table 1.** Summary statistics for the genome assembly of *Penstemon davidsonii*.

Total bases	437,568,744
Contig count	2,688
Largest contig	55,463,854
N50	40,949,504
L50	5
N90	50,886
L90	390
Ns	125
GC content	34.7%
BUSCO scores	C: 95.3% (S: 85.3%, D: 10%), F: 1.2%, M: 3.5%, *n*: 2326

BUSCO parameters are as follows: C, complete BUSCOs; S, complete and single-copy BUSCOs; D, complete and duplicated BUSCOs; F, fragmented BUSCOs; M, missing BUSCOs; and *n*, total BUSCO groups searched.

Our initial assembly was 613 Mb, made up of 9,237 contigs. Duplicate purging reduced this to 438 Mb across 4,811 contigs. Then, scaffolding using 141.6 million Hi-C read pairs further reduced the number of contigs to 2,688 (Table 1). In the final assembly, 8 contigs >20 mb make up >60% of the reference and likely represent the 8 expected chromosomes. Completeness, as assessed by our BUSCO analysis, was ∼95% (Table 1).

The assembled transcriptome consists of a total of 247,266,183 bases across 274,807 isoform “transcripts” with a 99% BUSCO score [C: 99.2% (S: 31.4%, D: 67.8%), F: 0%, M: 0.8%, *n*: 255; see Table 1 for abbreviations]. These transcripts clustered into 180,352 putative genes, of which 152,610 had a single isoform and 2,604 had 10 or more isoforms. Genome annotation using this transcriptome yielded slightly >18,000 gene models, of which slightly >17,000 were protein-coding genes (Table 2). We also identified 4,331 miRNAs and 901 tRNAs (Table 2).

**Table 2.** Summary statistics for the *Penstemon davidsonii* annotation.

Genes	18,199
Protein-coding genes	17,299
Exons	109,386
CDSs	108,061
Expressed sequence matches	41,594
Protein matches	417,305
miRNA	4,331
tRNAs	901

In our final genome assembly, we identified and masked 257,613,589 bases of repeat content, which represents 58.87% of the total sequence. This included 135,653 transposable elements, made up of mostly Ty1/Copia (14.93% of genome sequence) and Gypsy/DIRS1 (8.96% of genome sequence) LTR retroelements (Table 3).

**Table 3.** Summary of repetitive element content found in the *Penstemon davidsonii* genome assembly.

Element type	Number of elements	Percent of sequence
SINEs	0	0
LINEs	6,831	0.83
LTR retroelements	110,753	29.15
DNA transposons	18,069	1.74
Rolling circles	5,414	0.40
Small RNA	1,225	0.31
Satellites	2,380	0.12
Simple repeats	91,183	1.28
Low complexity	13,875	0.15
Unclassified	391,416	24.90

This assembled and annotated genome of *P. davidsonii* provides a needed resource for the *Penstemon* evolutionary biology community. To date, most evolutionary analysis of *Penstemon* species has relied primarily on phenotypic analysis (e.g. Castellanos *et al*. 2006), examination of patterns exhibited by a small number of genes (e.g. Wessinger and Rausher 2015), or examination of patterns discernible from reduced representation sequencing (e.g. Stone and Wolfe 2021). As more *Penstemon* genomes come online, however, these analyses can be extended to a genomic scale, and investigators will be able to examine the evolutionary characteristics of entire *Penstemon* genomes.

Nevertheless, there is still room for improvement of the *P. davidsonii* genome. In particular, constructing a chromosomal-level assembly from these scaffolds will be necessary for determining properties such as recombination rate variation across the genome (e.g. Smukowski and Noor 2011) and addressing issues such as the evolution of structural variants (e.g. Ostevik *et al*. 2020) and genomic patterns of introgression (e.g. Martin and Jiggins 2017).

## Data Availability

All raw genomic data (PacBio WGS, Hi-C, and RNA-seq) and the annotated reference genome produced for this study have been deposited into the NCBI Sequence Read Archive under the accession number PRJNA1010203. The version of the genome and annotation described in this paper is accession JAYDYQ010000000. The assemblies, annotation files, and scripts for analysis can also be found on Dryad (doi:10.5061/dryad.4f4qrfjjr).
